# Transbronchial biopsy is useful in predicting UIP pattern

**DOI:** 10.1186/1465-9921-13-96

**Published:** 2012-10-29

**Authors:** Sara Tomassetti, Alberto Cavazza, Thomas V Colby, Jay H Ryu, Oriana Nanni, E Scarpi, Paola Tantalocco, Matteo Buccioli, Alessandra Dubini, Sara Piciucchi, Claudia Ravaglia, Christian Gurioli, Gian Luca Casoni, Carlo Gurioli, Micaela Romagnoli, Venerino Poletti

**Affiliations:** 1 Department of Diseases of the Thorax, Via C. Forlanini, Forlì, FC, 34-47121, ITALY; 2 Department of Pathology, Arcispedale S Maria Nuova – IRCCS, Reggio Emilia, Italy; 3 Department of Pathology , Mayo Clinic, Scottsdale, AZ, USA; 4 Division of Pulmonary and Critical Care Medicine, Mayo Clinic, Rochester, MN, USA; 5 Biostatistics and Clinical Trials Unit, Istituto Scientifico Romagnolo per lo Studio e la Cura dei Tumori (IRST), Meldola, FC, Italy; 6 Department of Pathology, Forlì, Italy; 7 Department of Radiology, Forlì, Italy

**Keywords:** Bronchoscopy, Idiopathic pulmonary fibrosis, Interstitial lung diseases, Transbronchial biopsy, Usual interstitial pneumonia

## Abstract

**Background:**

Usual interstitial pneumonia (UIP), is a necessary feature pathologically or radiologically for the diagnosis of idiopathic pulmonary fibrosis (IPF). The predictive value of transbronchial biopsy (TBB) in identifying UIP is currently unknown. The objective of this study is to assess the accuracy with which histopathologic criteria of usual interstitial pneumonia (UIP) can be identified in transbronchial biopsy (TBB) and to assess the usefulness of TBBx in predicting a the diagnosis of UIP pattern. We conducted a retrospective blinded and controlled analysis of TBB specimens from 40 established cases of UIP and 24 non-UIP interstitial lung diseases.

**Results:**

Adequate TBB specimens were available in 34 UIP cases (85% of all UIP cases). TBB contained histopathologic criteria to suggest a UIP pattern (ie. at least one of three pathologic features of UIP present; patchy interstitial fibrosis, fibroblast foci, honeycomb changes) in 12 cases (30% of all UIP cases). Sensitivity, specificity, positive and negative predictive values for the two pathologists were 30% (12/40), 100% (24/24), 100% (12/12), 46% (24/52) and 30% (12/40), 92% (22/24), 86% (12/14), 55% (22/40) respectively. Kappa coefficient of agreement between pathologists was good (0.61, 95% CI 0.31-0.91). The likelihood of identifying UIP on TBB increased with the number and size of the TBB specimens.

**Conclusion:**

Although sensitivity is low our data suggest that even modest amount of patchy interstitial fibrosis, fibroblast foci, honeycomb changes detected on TBB can be highly predictive of a UIP pattern. Conversely, the absence of UIP histopathologic criteria on TBB does not rule out UIP.

## Introduction

Idiopathic Pulmonary Fibrosis (IPF) is a progressive and lethal form of interstitial lung disease (ILD) of unknown etiology [1,2]. It is important for the pulmonary physician to correctly diagnose IPF and separate it from other interstitial pneumonias that have a better prognosis and response to therapy. The international consensus [1] defined the presence of histological or radiological pattern of usual interstitial pneumonia (UIP) as a necessary criterion for the diagnosis of IPF. Surgical lung biopsy (SLB) provides the best tissue samples to distinguish UIP from other forms of idiopathic interstitial pneumonias and other processes that can mimic IPF [3,4]. However SLB is burdened by associated risks and costs and is not advisable in a large portion of the elderly and more severely compromised patients [5,6].

Recognition of UIP on transbronchial biopsy (TBB) could obviate the need for a surgical procedure. Ensminger et al. [7] reported in a large cohort of diverse ILD patients that TBB is “clinically” useful in 75% of procedures, and in approximately one-third of patients the procedure fail to obtain an adequate quantity of lung parenchyma. For UIP specifically, Berbescu et al. have suggested that TBB is useful in one third of cases. Nevertheless, current opinion generally militates against the use of TBB for diagnosing UIP, but some physicians still rely on TBB and bronchoalveolar lavage (BAL) in the evaluation of patients with suspected IPF, particularly in those at high surgical risk [8-11]. Two retrospective and unblinded studies, including that of Berbescu et al. showed that TBB specimens may display the histologic features of UIP [12,13]. Katzenstein recently reported their experience that, in the proper clinical context and with adequate tissue sampling, TBB can support a diagnosis of UIP in 43% of cases (9/22)”. [14].

It was within this context that we sought to determine the accuracy of TBB in diagnosing UIP.

## Materials and methods

This study was approved by the Area Vasta Romagna Ethical Committee (Prot 1493/2010 i.5 / 232). 64 patients with ILD between January 2001 and December 2008 were identified from the ILD database of Pneumology Unit at GB Morgagni Hospital, Forlì, FC, Italy. All patients underwent TBB within 12 months prior to SLB. These patients had clinical and radiologic features compatible with a fibrotic ILD, but insufficient elements to achieve a IPF diagnosis by current international consensus criteria. Bronchoscopists chose the lobe to biopsy taking into account the appearance and distribution of interstitial involvement on high resolution computed tomography (HRCT). Peripheral areas with predominant reticular involvement were selected regardless of the coexistence of other ancillary findings such as ground glass, discrete cysts or micronodules. Areas of honeycombing were avoided.

### Clinical assessment

We collected clinical data at the time of the first visit at our Institution, during which the diagnosis of ILD was established, and during follow-up. We extracted the following data: age, gender, past medical history, medications, smoking history, environmental exposure history, physical examination findings, laboratory results, BAL findings and pulmonary function data.

### Radiological assessment

HRCT images of 64 patients performed within 12 months prior to SLB were reviewed by one radiologist (SP). 1 or 1.5-mm collimation section were obtained at 10-mm intervals or volumetrically on multi-detector CT scanners with 0.6- or 1-mm collimation and 1-mm reconstruction. All images were de-identified and reviewed at window settings optimized for lung parenchyma (width, 1500–1600 HU; level, -500 to −600 HU). The radiologist defined the presence of definite UIP, possible UIP, or inconsistent with UIP according to the current guidelines [1].

### Pathologic assessment

Hematoxylin-eosin–stained slides from SLB and TBB were de-identified and sent to two pathologists (AC and TVC). The pathologists examined the pathologic specimens independently and in a blinded fashion. SLB specimens had been obtained from a single lobe in 9 patients and two lobes in 55 patients. One SLB representative slide per each lobe was provided for interpretation. All cases were finally reviewed as a complete patient and classified as definite UIP, probable UIP, possible UIP or non-UIP according to current guidelines [1] as best could be applied in the setting of TBB.

**Figure 1:**
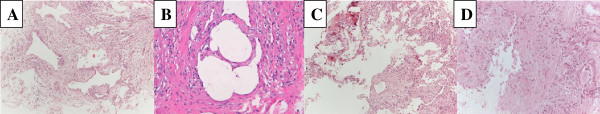
**Transbronchial biopsies in three different patients with a final diagnosis of UIP.A**) An area of scarring/honeycombing at the left, showing architectural abnormal lung with fibrosis and enlarged airspaces bordered by bronchiolar epithelium. Mucostasis is frequently present but can be absent, like here. Hematoxylin-Eosin, 100X. **B**) Higher magnification showing enlarged airspaces with bronchial metaplasia in the absence of mucus. Hematoxylin-Eosin, 20X. **C**) An area of fibrosis with a patchy character, in which collagen-type interstitial fibrosis is present adjacent to relatively normal alveoli. Hematoxylin-Eosin, 100X. **D**) A fibroblast focus, consisting in a small, dome-shaped collection of myofibroblasts within myxoid stroma, adjacent to fibrotic lung tissue. Fibroblast foci are frequently covered by hyperplastic pneumocytes, like here. Hematoxylin-Eosin, 200X.

One TBB hematoxylin-eosin–stained slide from each case was provided to the pathologists for interpretation. For analysis of the TBB data the following parameters were recorded: the site where TBB was performed, the number and diameter of TBB pieces, the number of fragments with alveolated lung parenchyma. At least one fragment of alveolated lung parenchyma was required to classify the biopsy as adequate. TBB was considered non-diagnostic when lacking histopathologic criteria sufficient to define a specific histopathologic pattern. In order to analyze whether histopathologic criteria of UIP were detectable on TBB, the presence or absence of three histopathologic criteria were assessed: honeycomb change, patchy fibrosis and fibroblast foci (including their number) (Figure 1). Patchwork pattern is characterized by alternating zones of abnormal and normal lung side by side without transition zones. Fibroblast foci are composed of small dome-shaped collections of spindleshaped fibroblasts and myofibroblasts within myxoid stroma. Honeycomb areas are characterized by enlarged airspaces with thickened walls and lined by bronchiolar epithelium and often filled by mucin and variable numbers of inflammatory cells. In the absence of honeycombing, pathologists considered mere architectural distortion to be nonspecific and insufficient for UIP diagnosis. The findings on TBB were considered compatible with UIP pattern when one or more of the three histopathologic criteria (honeycomb change, patchy fibrosis, fibroblast foci) were present, irrespective of the coexisting features suggesting the presence of acute lung injury including organizing pneumonia. All cases that showed pathological findings suggesting an alternative diagnosis were considered not-UIP.

### Definition of UIP

UIP was defined as the presence of UIP pattern on surgical lung biopsy. In cases of disagreement between the two pathologists, the final consensus diagnosis required review of histopathologic and radiologic findings along with clinical and follow-up data [1].

### Statistical methods

The primary outcome measures of this study were sensitivity and specificity of identifying histopathologic criteria of UIP (honeycomb change, patchy fibrosis and fibroblast foci) in TBB. Sensitivity and specificity of TBB in detection of UIP were calculated as the proportion of true positives of the total number with UIP and as the proportion of true negatives of the total number of non-UIP, respectively. The specificity of TBB in detection of UIP was compared to specificity of SLB diagnosis of UIP measured as the proportion of true negatives on TBB of the total number of patients without UIP and compared with the proportion of true negative on SLB of the total number of patients without UIP.

The interobserver agreement between pathologists was measured by K statistic and relative 95% confidence interval (95% CI).

Statistical analyses were carried out using SAS Statistical Software (version 9.1, SAS Institute, Cary, NC).

## Results

### Patient characteristics

Among 40 UIP cases 37 were “definite” IPF and 3 were “possible” IPF according to current guidelines [1]. The 37 cases of definite IPF included 36 cases with definite UIP on surgical SLB by at least one pathologist and possible UIP defined by HRCT, and one case of probable UIP read by both pathologists and possible UIP on HRCT. These 37 cases of possible UIP on HRCT presented reticular abnormalities with bibasilar and subpleural distribution in the absence of honeycombing. All three cases with a final clinical diagnosis of possible IPF had definite UIP on SLB by both pathologists and HRCT findings that were identified as inconsistent with UIP. Among cases with HRCT findings inconsistent with UIP 2 cases presented reticular abnormalities with upper lobe predominance and one case extensive ground glass. HRCT features of UIP did not correlate with UIP histological findings on TBB.

24 control (non-UIP) cases included 2 bronchiolitis, 2 chronic hypersensitivity pneumonia (HP), 2 fibrotic organizing pneumonia (OP) related to infections, 2 desquamative interstitial pneumonia, 3 chronic granulomatous lung diseases not otherwise specified, 2 Langerhans’ cell histiocytosis (LCH), 2 exogenous lipoid pneumonia, 6 nonspecific interstitial pneumonia (NSIP) (one cellular and five fibrotic), 2 sarcoidosis and 1 primary pulmonary hypertension (PAH). In four cases (fibrotic-NSIP, chronic HP, PAH, fibrotic-OP) pathologists did not reach a final consensus diagnosis on SLB. For these four difficult cases final diagnosis relied on consensus after review of clinical radiological and pathological along with follow-up data.

### Transbronchial biopsy in the Diagnosis of UIP and non-UIP Patterns

Characteristics of the TBB specimens are shown in Table 1.

**Table 1:** Characteristics of transbronchial biopsy specimens

	**TOTAL n = 64**	**UIP n = 40**	**Other ILDs n = 24**
TBB specimens			
Adequate biopsies, n of cases (%)	56 (87.5)	34 (85)	22 (92)
Number of alveolated pieces, median (range)	3 (1–8)	3 (1–7)	3 (1–8)
Size of alveolated pieces in mm, median (range)	2.34 (1.0-5.8)	2.15 (1.0-4.3)	2.60 (1.1-5.8)
Diagnostic biopsies (pathologist 1) n of cases (%)	16 (25)	12 (30)	4 (16)
Diagnostic biopsies (pathologist 2) n of cases (%)	15 (23)	12 (30)	3 (12.5)
Site of TBB			
Upper lobe, n (%)	12 (19)	6 (15)	6 (25)
Middle lobe or lingula, n (%)	3 (5)	1 (2.5)	2 (8)
Lower lobe, n (%)	49 (77)	33 (82.5)	16 (66)
Cases with TBB and SLB taken in the same lobe, N (%)	43 (67)	29 (72)	14 (58)

Among diagnostic TBB cases the first pathologist correctly identified non-UIP in four out of twenty-four TBBs (16%), whereas all twelve TBBs (100%) considered consistent with UIP were confirmed UIP on SLB. The second pathologist correctly identified non-UIP cases in three out of twenty-four TBBs (12.5%), and twelve out of fourteen TBBs considered consistent with UIP (85%) were confirmed as UIP. Even thought the TBB yielded adequate specimens in a high portion of cases (92%), only one third of TBB specimens of non-UIP cases were sufficient to detect a histopathologic pattern and among these only in a minority (12.5% and 16%) was identified the correct diagnosis. Characteristics of TBB specimens of non-UIP cases are reported in Table 2.

**Table 2:** Transbronchial lung biopsy analysis in non-UIP ILD Cases

	**Total number of cases**	**PATHOLOGIST #1 diagnostic cases**	**PATHOLOGIST #2 diagnostic cases**
non-UIP cases, N (%)	24	4 (16)	3 (12.5)
NSIP, N (%)	6	1 (16)	2 (33)
DIP, N (%)	2	1 (50)	0
Chronic HP, N (%)	2	0	0
LCH, N (%)	2	0	0
Fibrotic OP, N (%)	2	1 (50)	0
Bronchiolitis, N (%)	2	0	0
Fibrotic sarcoid, N (%)	2	0	0
Chronic granulomatosis NOS, N (%)	3	1 (33)	1 (33)
Lipoid pneumonia, N (%)	2	0	0
PAH, N (%)	1	0	0

*Definitions of abbreviations*: *HP* hypersensitivity pneumonia, *OP* organizing pneumonia, *DIP* desquamative interstitial pneumonia, *NOS* not otherwise specified, *LCH* Langerhans’ cell histiocytosis, *PAH* primary pulmonary hypertension.

TBBs which displayed histopathologic criteria of UIP (n=12) showed a significantly higher number and larger diameter of tissue fragments compared to UIP cases (by SLB) whose TBBs were read as adequate but non diagnostic (n=22). Median number of fragments was 3.5 (range 1–7) for the former and 1 (range 1–5) for the latter, p= 0.003. Median diameter of the largest TBB specimen was 2.45mm (range 1.5-4.3) for the former and 1.75mm (range 1.0-2.8) for the latter, p=0.005.

The majority of TBB were performed in the lower lobes (49 cases, 77%) and in the same lobe as SLB in 43 cases (67%).

### TBB accuracy for prediction of UIP pattern

The pathologic findings are summarized in Table 3.

**Table 3:** Transbronchial lung biopsy analysis in usual interstitial pneumonia cases

	**PATHOLOGIST #1 40 UIP CASES**	**PATHOLOGIST #2 40 UIP CASES**
DIAGNOSTIC OF UIP, N (%)	12 (30)	12 (30)
Three criteria, N (%)	0	1 (2.5)
Two out of three criteria, N (%)	4 (10)	9 (22.5)
One out of three criteria, N (%)	8 (20)	2 (5)
OTHER ILDs , N (%)	8 (20)	8 (20)
INTEROBSERVER AGREEMENT for the presence of at least one feature of UIP (Kappa )	0.61 (95% CI 0.31-0.91)	
INTEROBSERVER AGREEMENT for the presence of the same number of UIP features (Kappa)	0.46 (95% CI 0.28-0.62)	

Among 40 cases of established UIP, the first pathologist detected pathologic features compatible with UIP in 12 (30%) TBB specimens. Eight cases (20%) were considered consistent with alternative diagnoses: acute lung injury (6 cases) and desquamative interstitial pneumonia/respiratory bronchiolitis-ILD (2). Sensitivity and specificity of TBB in detection of UIP features were 30% (12/40) and 100% (24/24), respectively. Positive and negative predictive values were 100% (12/12) and 46% (24/52), respectively.

Among the 40 established UIP cases, the second pathologist detected UIP features in 12 (30%) TBB specimens. Eight cases (20%) were considered consistent with an alternative diagnosis: acute lung injury in all 8. Sensitivity and specificity of TBB in detection of UIP were respectively 30% (12/40) and 92% (22/24). Positive and negative predictive values were 86% (12/14) and 55% (22/40), respectively.

### TBB accuracy for detection of UIP criteria

In only one case did one of the two pathologists identify all three pathologic criteria of UIP . Both pathologists identified ten TBB cases showing two of three histopathologic criteria of UIP. All ten cases were confirmed to be UIP, with a positive predictive value for both pathologists of 100% (4/4 and 9/9, respectively). In ten TBB cases both pathologists identified only one of three histopathologic criteria of UIP. Eight out of these ten cases were confirmed to be UIP. Positive predictive value of the two pathologists was 100% (8/8) and 50% (2/4), respectively. The two false positive cases are described in Table 4. Analyzing the pathologic features of UIP separately it appears that honeycombing is the feature better recognized by the two pathologists with a good Kappa coefficient of agreement (0.64). Agreement was moderate (K coeff 0.48) on recognition of fibroblast foci and fair (K coeff 0.29) on recognition of patchy fibrosis.

**Table 4:** Summary of non-UIP cases in which TBB detected UIP features

**Final diagnosis**	**SLB**	**PATHOLOGIST #1 TBB findings**	**PATHOLOGIST #2 TBB findigs**
Fibrotic OP related to chronic infection	OP, DAD, suppurative foci.	OP	Patchy interstitial fibrosis
Fibrotic iNSIP	Fibrotic NSIP	Non-patchy interstitial fibrosis	Patchy interstitial fibrosis

*Definitions of abbreviations*: *SLB* surgical lung biopsy, *OP* organizing pneumonia, *NSIP* non specific interstitial pneumonia.

### Interobserver agreement

Interobserver agreement between the two pathologists in recognition of UIP (i.e. identification of one or more UIP features) on TBB was good, with a simple Kappa coefficient of 0.61 (95% CI 0.31-0.91). Interobserver agreement on SLB diagnosis of UIP was very good (0.82, 95% CI 0.67-0.97). Interobserver agreement in the recognition of the presence of the same number of pathologic features of UIP measured as simple Kappa coefficient was moderate for both TBB and SLB (0.46, 95% CI 0.28-0.62 for TBB and 0.51, 95% CI 0.37-0.67 for SLB).

### TBB safety

We observed five cases (8%) of pneumothorax (one required chest tube drainage) and one case (2%) of pneumomediastinum. Four of these cases were UIP, one DIP and one sarcoidosis. No correlation was observed with the number of TBB specimens. The median number of fragments was 5 (range 2–6) in patients with complications, compared to 5 (range 1–12) in cases without complications.

## Discussion

This study shows that TBB specimens can yield some of the findings of a UIP pattern in a portion of such patients. Two provocative findings emerge from this study. First, TBB is a useful mini-invasive tool that can identify some of the UIP criteria in about one-third of cases with a high positive predictive value and a good interobserver agreement. Second, TBB is less useful in diagnosing other fibrotic infiltrative diseases that can mimic IPF.

Our data confirm those previously published by Berbescu and co-workers [12] demonstrating that TBB specimens contain some (or all) of the histopathologic criteria of UIP in approximately one third of patients. Diagnostic biopsies contained more fragments and larger amount of alveolated lung parenchyma compared to UIP cases where TBB did not display UIP criteria, but even in cases with only one adequate fragment it was possible to recognize some features of UIP. One pathologist was able to detect all three features of UIP in a case with only one alveolated fragment. This shows that a very experienced pathologist can sometimes reach a confident diagnosis of UIP even with a minimal biopsy specimen. In the presence of two or more histopathologic criteria of UIP seen on TBB, the diagnosis of UIP is always confirmed by SLB. These observation supports the view that even the pathologic findings on small samples in the appropriate clinical and radiologic setting can be highly predictive of a UIP pattern. The aim of this study was limited to defining the accuracy with which histopathologic criteria of UIP can be identified in TBB. It should be recognized, however, that numerous lung diseases can manifest histological pattern of UIP and the use of multidisciplinary approach is mandatory for the final diagnosis. Therefore, further studies are needed to evaluate the value of adding TBB results to clinical and radiological findings in order to define the impact of TBB on diagnostic confidence in the multidisciplinary diagnosis of IPF and other ILDs.

The accuracy of TBB is mostly limited by its low negative predictive value and by its low sensitivity which relate to a high portion of inadequate and non diagnostic fragments. Number and size of fragments strongly influenced the accuracy of TBB in diagnosing UIP. We can hypothesize that the use of newer bronchoscopic biopsy methods, such as cryoprobes, may yield larger specimens that can highly improve the diagnostic yield [15]. TBB in UIP cases was more informative than in non-UIP cases.

We acknowledge that the limitations of this study include the relatively small number of cases and that this study was not designed to evaluate the accuracy of TBB in diagnosing non-UIP ILDs. Therefore we are not able to draw any firm conclusion on the diagnostic utility of TBB in this other group of diseases. However, it is interesting to note that TBB was not useful to discriminate some of the ILDs that commonly enter the differential diagnosis of IPF, such as DIP, fibrotic NSIP and chronic HP [17,18]. It has been described that in some patients non-UIP patterns, such as RB-ILD, DIP and NSIP, can coexist with UIP [19,20] and diagnosis can be rather challenging in such cases. Our study report similar findings for TBB showing that fibrosing ILDs other than UIP present complex pathologic features that are rarely detectable by TBB and should be regarded as non diagnostic in the context of a multidisciplinary diagnosis of IPF. On the other hand UIP pattern can occur in diseases that can mimic IPF such as chronic HP, chronic sarcoidosis, asbestosis. In these cases TBB findings can be misleading. To reach the correct final diagnosis the interpretation of the UIP pattern requires a careful multidisciplinary evaluation.

For individual histological criteria of UIP evaluated on TBB we found a fair agreement between the two pathologists. Nicholson et al. [20,21] reported levels of agreement between pathologists for individual histological criteria of UIP in SLB to be higher (mean K 0.75 for fibroblast foci and 0.76 for honeycombing) than ours that are based on TBB specimens.

We acknowledge several limitations in the retrospective, mono-centric design of this study and in the limited number of cases and controls examined. The blinded pathologic review method used represent another limit of this study. Only one slide per sample was reviewed for both SLB and TBB and special stains were not used. Ideally multiple levels should be evaluated but they were not available in all our cases. In real practice the use of deeper levels and special stains could be useful to reveal pathologic features not present in only one haematoxylin eosin stained slide. This limit the opportunity to test true diagnostic performance of TBB in a process more closely resembling standard practice and emphasize the bias that pathologists involved are experts. In current practice more than one slide per case should be evaluated and special stains are recommended in selected cases. The best study design would be a controlled, blinded, prospective study using multiple slides and special stains. However, due to the complete lack of controlled and blinded data on this topic, we postulated that this was a mandatory preliminary step. TBB were obtained in a referral centre for ILDs by experienced bronchoscopists and also this might have influenced the results. However the number and size of alveolated pieces does not differ from previous reports and is in line with feasibility data reported by others [12,13].

## Conclusion

In summary, we conclude that TBB can diagnose UIP in a portion of such patients. In the appropriate clinical-radiological setting, TBB showing at least two histopathological criteria of UIP reliably diagnoses UIP pattern. Even for cases with only one criterion TBB appears to be informative, but with a lower predictive value.

## Competing interests

The authors declare that they have no competing interests.

## Authors’ contributions

ST, JHR and VP were major contributors in designing and writing the manuscript. TVC and AC performed the histological examination. ON, ES, MB and PT analyzed the patient data. SP performed the radiological revision of cases. CR, CG, GLC, MR and AD collected and interpreted patients data. All authors read and approved the final manuscript.
